# MRI of diffuse-type tenosynovial giant cell tumour in the knee: a guide for diagnosis and treatment response assessment

**DOI:** 10.1186/s13244-023-01367-z

**Published:** 2023-02-01

**Authors:** Geert Spierenburg, Carlos Suevos Ballesteros, Berend C. Stoel, Ana Navas Cañete, Hans Gelderblom, Michiel A. J. van de Sande, Kirsten van Langevelde

**Affiliations:** 1 Department of Orthopaedic Surgery, Leiden University Medical Centre, Postzone J11-R-70, PO Box 9600, 2300 RC Leiden, The Netherlands; 2 Department of Radiology, Hospital Universitario Ramón y Cajal, Madrid, Spain; 3 Division of Image Processing, Department of Radiology, Leiden University Medical Centre, Leiden, The Netherlands; 4 Department of Radiology, Leiden University Medical Centre, Leiden, The Netherlands; 5 Department of Medical Oncology, Leiden University Medical Centre, Leiden, The Netherlands

**Keywords:** Tenosynovial giant cell tumour, Diffuse-type TGCT, Magnetic resonance imaging, Colony-stimulating factor 1, 3D segmentation

## Abstract

Tenosynovial giant cell tumour (TGCT) is a rare soft-tissue tumour originating from synovial lining of joints, bursae and tendon sheaths. The tumour comprises two subtypes: the localised-type (L-TGCT) is characterised by a single, well-defined lesion, whereas the diffuse-type (D-TGCT) consists of multiple lesions without clear margins. D-TGCT was previously known as pigmented villonodular synovitis. Although benign, TGCT can behave locally aggressive, especially the diffuse-type. Magnetic resonance imaging (MRI) is the modality of choice to diagnose TGCT and discriminate between subtypes. MRI can also provide a preoperative map before synovectomy, the mainstay of treatment. Finally, since the arrival of colony-stimulating factor 1-receptor inhibitors, a novel systemic therapy for D-TGCT patients with relapsed or inoperable disease, MRI is key in assessing treatment response. As recurrence after treatment of D-TGCT occurs more often than in L-TGCT, follow-up imaging plays an important role in D-TGCT. Reading follow-up MRIs of these diffuse synovial tumours may be a daunting task. Therefore, this educational review focuses on MRI findings in D-TGCT of the knee, which represents the most involved joint site (approximately 70% of patients). We aim to provide a systematic approach to assess the knee synovial recesses, highlight D-TGCT imaging findings, and combine these into a structured report. In addition, differential diagnoses mimicking D-TGCT, potential pitfalls and evaluation of tumour response following systemic therapies are discussed. Finally, we propose automated volumetric quantification of D-TGCT as the next step in quantitative treatment response assessment as an alternative to current radiological assessment criteria.

**Supplementary Information:**

The online version contains supplementary material available at 10.1186/s13244-023-01367-z.

## Key Points

- TGCT is categorised according to its site of involvement and growth pattern.
- D-TGCT is characterised by irregular synovial proliferation infiltrating multiple synovial knee recesses.
- Systematic MRI approach is provided for preoperative and systemic treatment response assessment.
- An overview of synovial knee recesses guides the radiologist in assessing blind spots.
- Machine learning-based tumour segmentation may be applied to assess D-TGCT tumour volume in the near future, so that treatment response assessment can be quantified and improved by artificial intelligence.

**Supplementary Information:**

The online version contains supplementary material available at 10.1186/s13244-023-01367-z.

## Introduction

Tenosynovial giant cell tumour (TGCT) is a fibro-histiocytic soft-tissue tumour that involves anatomical structures covered by a synovial membrane (joints, bursae and tendon sheaths). It can also affect extra-synovial locations, such as subcutaneous and intramuscular lesions [1]. Common symptoms are pain, swelling, stiffness, and limited function, leading to decreased quality of life [2].

TGCT can be classified according to its site (intra- and extra-articular) and growth pattern [3]. In the 2013 World Health Organisation classification of soft tissue and bone tumours, localised-type (L-TGCT) and diffuse-type (D-TGCT) replaced the terminology “giant cell tumour of the tendon sheath” and “pigmented villonodular synovitis”, respectively [1]. There is no clear histological distinction between both subtypes, therefore, diagnosis is based on radiological diagnosis and clinical presentation [4]. TGCT is a rare neoplasm with incidence rates of 45 and 5 per million person-years for L-TGCT and D-TGCT, respectively. TGCT has a female predilection (♀:♂; 2:1) and affects a relatively young patient group mainly aged between 30 and 50 years, although it can occur at any age [5, 6].

L-TGCT occurs in an extra-articular location in 90% of cases, involving tendon sheaths of the volar aspect of fingers (85%), followed by foot and knee locations (15%) [7]. These tumours primarily present as painless soft-tissue masses without joint dysfunction. The diffuse type originates predominantly in the intra-articular space of large joints such as the knee (70%) followed by the hip (15%) but often extends extra-articular [5]. The extra-articular D-TGCT form is mostly a secondary extension of intra-articular disease [8]. D-TGCT tends to present with chronic joint pain and swelling, often with progressive secondary osteoarthritis [2, 9]. D-TGCT represents a monoarticular disease, which means that in case of polyarticular involvement with similar MRI appearance, other diagnoses should be considered, such as gout, haemophilic or amyloid arthropathy.

TGCT subtypes share a common underlying pathogenesis, mainly related to a Colony-Stimulating Factor 1 (*CSF1*) translocation resulting in CSF1 overexpression. CSF1 overexpression causes an increase in neoplastic cells by binding to CSF1-receptors (CSF1R) and accumulating CSF1R presenting cells [10]. Histologically, TGCT shows an infiltrative growth pattern and comprises mononuclear cells, multinuclear osteoclast-like giant cells, macrophages and stromal hyalinization. Also, hemosiderin depositions are frequently observed [1].

MRI is the imaging modality of choice for diagnosing and evaluating disease severity [11]. It gives insight into areas that are not amenable for arthroscopic evaluation. Thereby, MRI can provide a preoperative map of D-TGCT localisations to evaluate common blind spots before open synovectomy [8]. Achieving complete resection can be challenging, especially in extensive tumour growth. Incomplete resections are associated with a higher chance of tumour relapse [12, 13]. Other treatment modalities such as radiosynoviorthesis or external beam radiotherapy have been used to reduce relapse rates. However, evidence regarding the efficacy of these treatments is scarce [14, 15]. Furthermore, the role of radiotherapy for TGCT remains controversial because it may result in complications such as osteoarthritis in a young patient population [13]. With the arrival of CSF1R-inhibitors, a novel systemic therapy for D-TGCT patients not amenable to surgery, MRI is essential to select and follow-up on target lesions [16, 17].

In this educational review, we demonstrate the imaging features of D-TGCT and highlight blind spots and potential pitfalls on MRI.

## Imaging features of D-TGCT

### Radiography

Conventional radiography provides a first modality to assess osteoarticular complaints of the knee. Radiographs of the knee in D-TGCT are often normal, although features of osteoarthritis may be present, such as osteophytes, joint space narrowing and subchondral sclerosis. Pressure erosions may occur on both articular joint surfaces in advanced stages, especially in joints with limited volume and joint space, such as the ankle, hip and shoulder [9]. In the knee, erosions have been described on radiographs in up to 30% of patients [18].

The presence of (peri)articular soft-tissue calcifications pleads against the diagnosis of D-TGCT and differential diagnoses such as gout or synovial chondromatosis should be considered [19].

### Ultrasound

Ultrasound is not part of the standard diagnostic workup of D-TGCT; however, it can be helpful in performing image-guided biopsies [20]. Appearance of D-TGCT has been described as hypoechoic irregular synovial thickening along with heterogeneous joint effusion and hyperemia, although these findings are non-specific and may be found in other types of diffuse synovitis [21]. In addition, ultrasound does not provide the necessary information and correct evaluation of the areas that should be carefully scrutinised and reported when evaluating D-TGCT.

#### MRI

MRI is the modality of choice to diagnose D-TGCT. The scanning protocol applied at our tertiary referral centre for bone and soft tissue tumours (3 Tesla, Ingenia, Philips, Eindhoven, The Netherlands) is shown in Additional file 1: Table S1. A gradient-echo sequence may be beneficial for detecting hemosiderin related to tumour bleeding. Intravenous gadolinium contrast aids in tumour detection and is helpful for follow-up after synovectomy. TGCT can present with variable MRI appearances given its heterogeneous histological composition and the variety in growth patterns (intra- and/or extra-articular) [3].

D-TGCT findings include irregular synovial thickening (> 5 mm), typically described as “frond-like” with villous or nodular morphology [3]. This synovial proliferation tends to engulf associated reactive joint effusion resulting in multiloculated thick-walled trapped cystic masses, especially seen in the subgastrocnemius synovial recesses and Baker’s cysts [18].

**Fig. 1:**
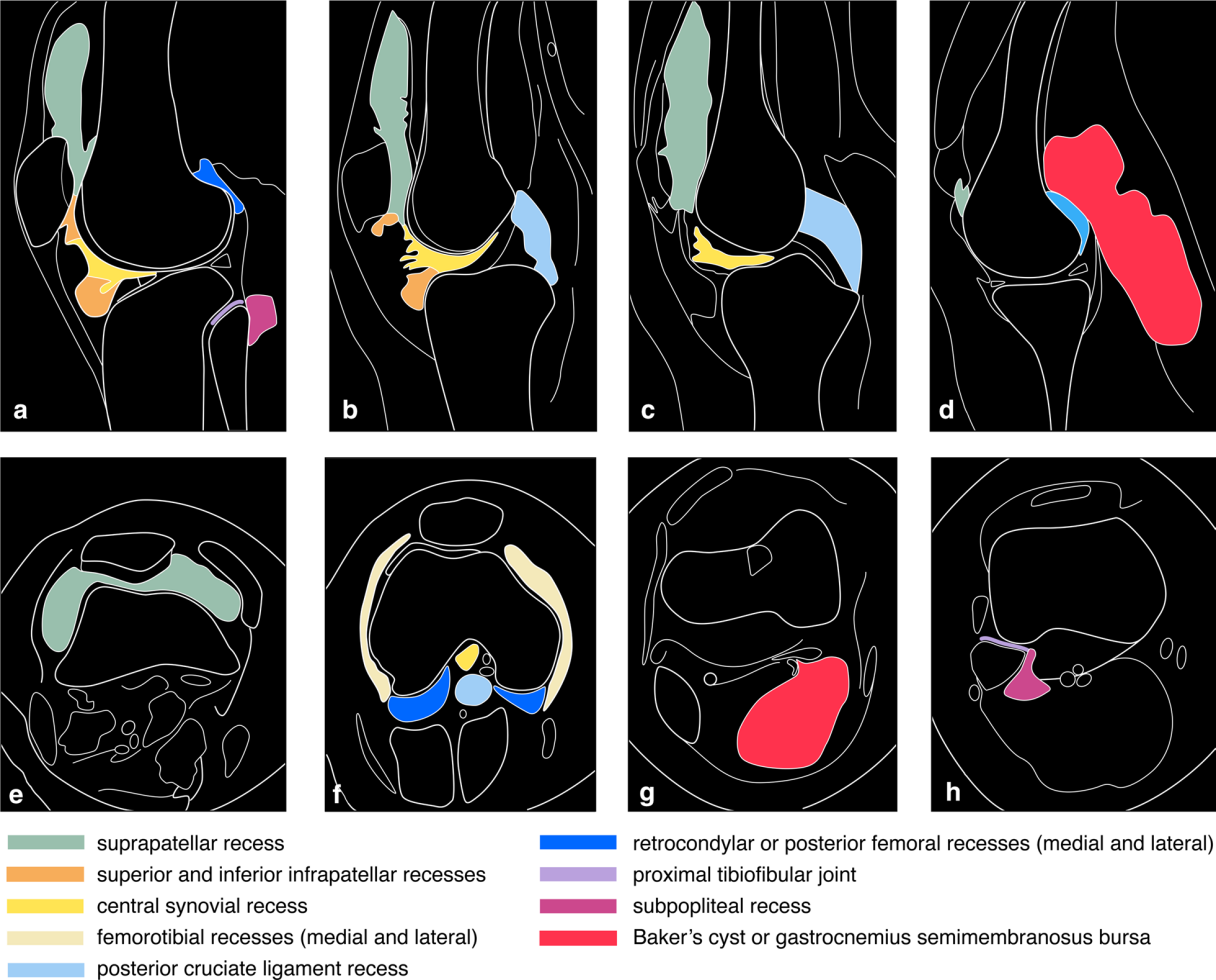
Schematic overview of synovial recesses in the knee. **a**–**d** Sagittal drawings from lateral (**a**) to medial (**d**). **e**–**h** axial drawings from superior (**e**) to inferior (**h**)

D-TGCT intra-articular forms are likely to spread diffusely, developing a multicompartmental growth pattern involving at least two contiguous intra-articular synovial recesses. In the knee, several recesses may be involved, as illustrated in the detailed description of Fig. 1.

D-TGCT’s extra-articular growth pattern mainly occurs secondary to intra-articular extension through transcapsular fenestrations [8]. Mastboom et al. defined extra-articular extension as TGCT involvement outside the synovial lining of the joint. Furthermore, cartilage invasion, cortical bone erosions, muscular/tendinous, ligament and neurovascular involvement were proposed as parameters that determine the severity of D-TGCT [22]. In the knee, tibial nerve encasement is rare but may be symptomatic. D-TGCT may extend into femoral and tibial medullary tunnels in patients with anterior cruciate ligament (ACL) reconstruction.

**Fig. 2:**
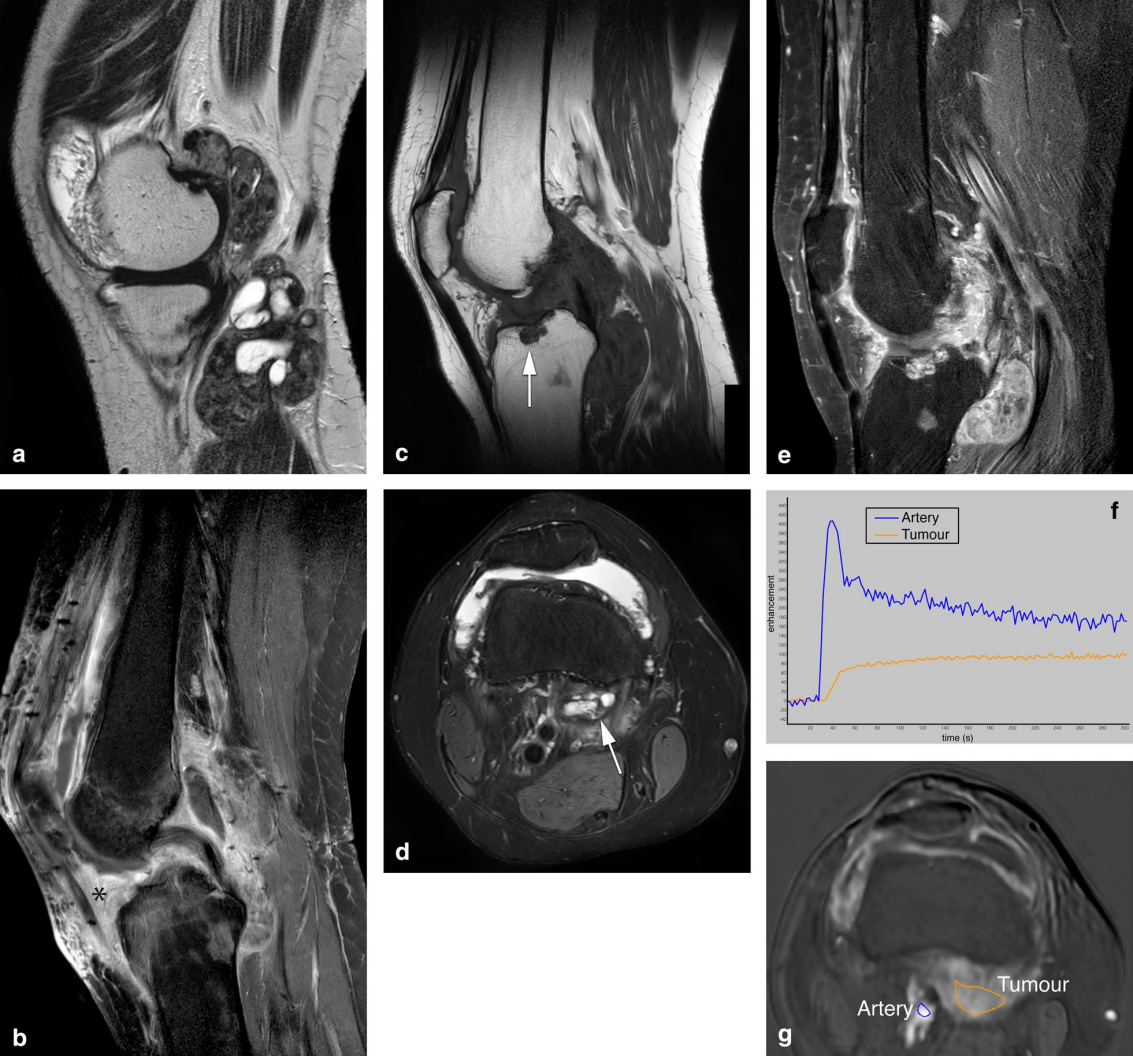
A case of D-TGCT demonstrating pre and post synovectomy findings on MRI. **a** Sagittal T2 weighted image shows multilobular posterior tumour with low signal intensity, and adjacent cyst-like components present within tumour in the popliteal cyst. Anterior, in the medial gutter of the suprapatellar recess smaller synovial proliferations are present. **b** Sagittal T1 SPIR post contrast performed 3 months after anterior and posterior synovectomy shows surgical clips with metal artefact anterior and posterior in the soft tissues, thickening of the quadriceps tendon, subcutaneous oedema and marked enhancement in Hoffa’s fatpad (asterisk) and along the posterior cortex of the tibia (subpopliteal recess). This mass-like enhancement can be post operative but residual tumour cannot be excluded at this time. *MRI performed 3 years post synovectomy:* **c** Sagittal T1 shows a bone erosion centrally in the tibial plateau (arrow). Furthermore, soft tissue masses posteriorly in the knee are present containing foci of low signal intensity. **d** Axial PD SPAIR shows a typical location of a lesion containing cystic components at the medial retrocondylar recess (arrow). **e** Sagittal T1 SPIR post contrast demonstrates enhancement of tumour within the tibia plateau erosion and of the posterior mass lesions. Note that Hoffa’s fat pad shows normalisation of fatty signal intensity except for a rim of tumour enhancement in the central synovial recess and inferior infrapatellar recess. **f**, **g** Time intensity curve of the tumour based on the region of interest (orange line) of the lesion demonstrated in **d**, showing early enhancement within 10 s after the artery (blue line) followed by a plateau phase (type III curve suggestive of a benign lesion)

**Fig. 3:**
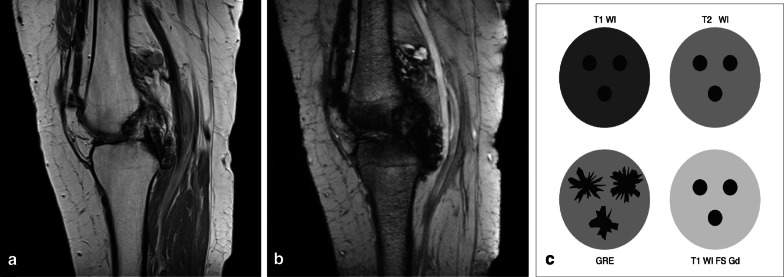
Blooming artefact. **a** Sagittal PD-weighted MR image of the knee in a patient with D-TGCT demonstrates multiple low signal intensity synovial lesions posterior to the PCL, along the posterior cortex of the femoral metaphysis and in the collapsed suprapatellar recess. **b** Sagittal T2- gradient echo weighted MR image of the knee showing blooming artefact: the low signal intensity synovial lesions containing hemosiderin increase in size and are ill defined, appearing as cloud-like dark areas. **c** Schematic illustration of hemosiderin signal intensities on gradient echo (GRE) weighted sequences versus T1- and T2-weighted sequences. Gradient echo images show increased size of the hemosiderin foci with irregular margins, this is called “blooming”

D-TGCT signal intensity is heterogeneous. T1-weighted imaging (T1-WI) shows a hypointense to iso-intense signal, whereas fluid-sensitive sequences show a hyperintense signal with foci of low signal intensity corresponding to hemosiderin in the tumour (Fig. 2). D-TGCT is prone to bleeding (bleeding is more common than in L-TGCT), and therefore hemarthrosis is a common finding expressed as low signal intensity on both T1-WI and fluid-sensitive sequences. Haemorrhage constitutes a classic D-TGCT imaging hallmark mostly detected as blooming on gradient echo (GRE) images. This imaging feature has been described as pathognomonic to suggest TGCT diagnosis, but its absence does not exclude TGCT [23]. Blooming is a paramagnetic susceptibility artefact secondary to hemosiderin deposition defined as enlargement and disproportionately lower signal intensity of blood deposits on GRE images compared to spin-echo (SE) sequences (Fig. 3). Scout GRE acquisitions should be employed cautiously in the search for blooming owing to high false-negative rates [23]. Diffusion-weighted images (DWI) can be deceptive because TGCT (both localised and diffuse subtypes) depict intrinsically low apparent diffusion coefficient (ADC) values due to hemosiderin deposits [24]. D-TGCT shows avid heterogeneous enhancement. From dynamic post-contrast imaging (perfusion), a time-intensity curve can be obtained showing rapid early enhancement with a plateau phase (Fig. 2) [25].

Areas of intralesional fat with high signal intensity on T1-WI related to deposition of lipid-laden macrophages (xanthoma cells) are a classic feature of D-TGCT, however, this finding is uncommon [3, 23]. This feature may represent entrapped fronds of perisynovial fat or subsynovial fat metaplasia as a reactive process to chronic TGCT, similar to lipoma arborescens in rheumatoid or psoriatic arthritis.

## Differential diagnoses

**Fig. 4:**
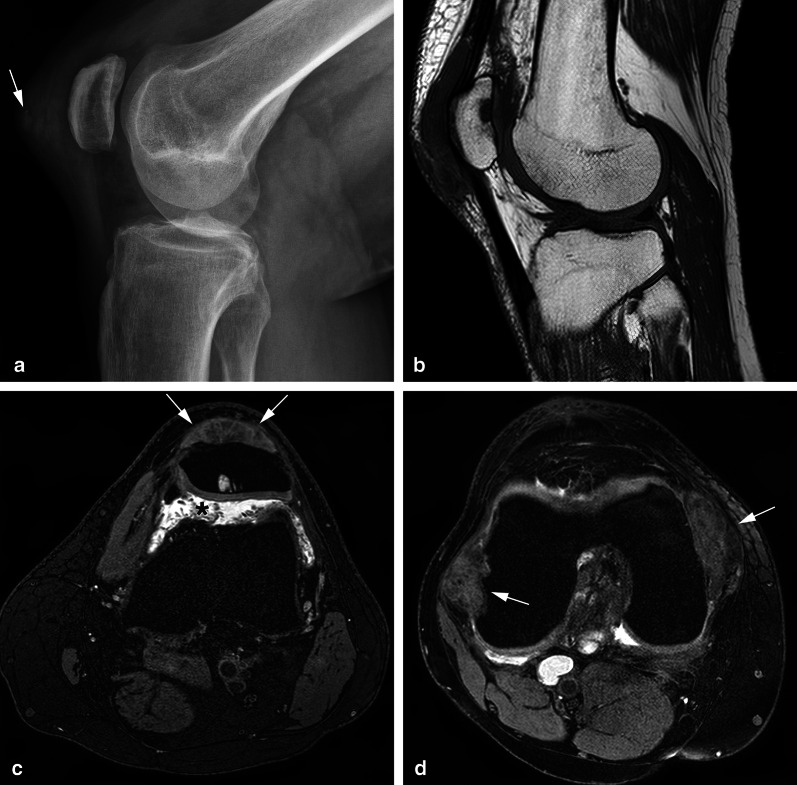
Differential diagnosis: gout in the knee. **a** Lateral radiograph demonstrates marked pre-patellar soft tissue swelling containing increased density and several ill-defined calcifications (arrow). **b** Sagittal T1 shows a prepatellar, low signal intensity oval shaped soft tissue mass and a subchondral cyst in the patella. **c** Axial T2 FS confirms the prepatellar, low signal intensity mass invading the quadriceps tendon (arrows) and shows joint effusion containing multiple small synovial proliferations in the suprapatellar recess (asterisk). Aspiration of joint fluid with crystals confirmed the diagnosis of gout. **d** Axial T2 FS demonstrates low signal intensity soft tissue lesions in keeping with gout tophi deep to the collateral ligaments, causing erosion of the medial and lateral femoral condyles (arrows)

Gout tophi can present as both intra- and extra-articular or peri-articular nodules. Typical locations such as the subcutaneous fat or within the distal quadriceps or proximal patellar tendon can help distinguish gout from D-TGCT [26]. Gout and amyloidosis are soft-tissue masses appearing hypointense on T2 sequences, which can be intra-articular, mimicking TGCT (Fig. 4) [27]. Radiographs may be helpful to assess soft tissue calcifications, and dual-energy computed tomography (CT) may be performed to prove the presence of monosodium urate crystals in gout [28].

**Fig. 5:**
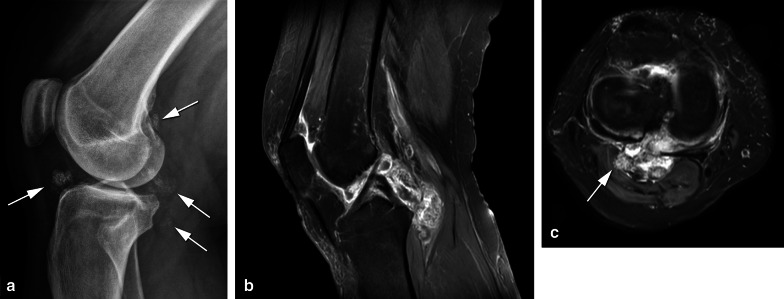
Differential diagnosis: synovial chondromatosis in the knee. **a** Lateral radiograph illustrating multiple punctiform masses in the soft tissues of the knee containing speckled calcifications (arrows). These calcifications are present in Hoffa and posterior in the knee. **b** Sagittal T1 SPIR post contrast showing rim enhancement of the synovial lesions, which contain low signal intensity foci corresponding to the calcifications on X-ray. Only minimal joint effusion is present surrounding the cruciate ligaments with rim enhancement. **c** Axial T2 DIXON shows posterior extracapsular extension into the lateral head of gastrocnemius muscle (arrow)

Synovial chondromatosis can present either as multiple round bodies similar in size and shape with a “snowstorm” or “cobblestone” pattern and a variable degree of calcification (85% is calcified) or coalesce into multiple intra-articular synovial masses (Fig. 5). The presence of calcification or metaplastic cartilage excludes the diagnosis of TGCT [19].

**Fig. 6:**
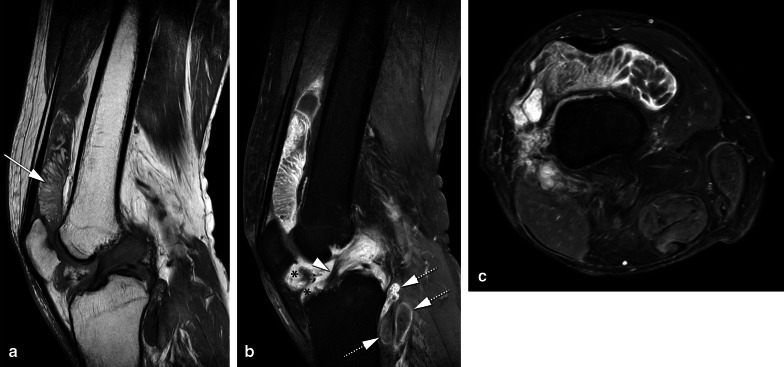
Differential diagnosis: lipoma arborescens. **a** Sagittal T1 demonstrates a hyperintense soft tissue mass in the suprapatellar recess, containing multiple villous proliferations (arrow). **b** Sagittal T1 SPIR post contrast shows the signal of the villi is suppressed and rim enhancement is present. In addition, there is mucoid degeneration of the anterior cruciate ligament and enhancing synovitis in the central synovial recess (arrowhead), superior and inferior infrapatellar recesses (asterisks), PCL and subpopliteal recess (arrows). **c** Axial T2 DIXON confirms fat suppression of the villi in the suprapatellar recess. These findings are in keeping with a lipoma arborescens, which is not a true neoplasm but rather a reactive process associated with rheumatoid or psoriatic arthritis or osteoarthritis

Lipoma arborescens is a chronic, slow-growing, intra-articular condition of benign nature, characterised by villous proliferation of the synovium with replacement of subsynovial connective tissue by mature fat cells. It may be misdiagnosed as TGCT if encountered on fat-suppressed fluid-sensitive sequences and not correlated with a native T1-weighted sequence. However, its feathery subsynovial fat deposition appearance on axial MR images is characteristic (Fig. 6) [29]. The classic location is the suprapatellar recess of the knee joint. In most cases, lipoma arborescens does not extend to other recesses, except when it develops in patients with chronic synovitis, such as in rheumatoid or psoriatic arthritis.

Synovial haemangioma may cause repetitive spontaneous hemarthrosis within a joint and thereby mimic D-TGCT clinically. However, the MRI appearance with a “bag of worms” caused by serpentine vascular channels and the presence of interspersed fat in a haemangioma may be helpful to distinguish the two entities. In addition, the enhancement pattern differs and a dynamic post contrast sequence can be used to determine slow-flow versus high-flow vascularity in the synovial haemangioma [30]. Resection is the treatment of choice.

Haemophilic arthropathy may mimic D-TGCT due to widespread hemosiderin depositions and often severe secondary osteoarthritis; however, the clinical history is usually straightforward and fatty components are absent due to a lack of foam cells [18].

## Treatment assessment in the knee

### Pre- and postoperative MRI findings

Surgery is the mainstay of TGCT treatment, performed either open or arthroscopically [31]. L-TGCT resection is relatively straightforward, with low recurrence rates (4–30%) controlled by re-excision [32]. On the other hand, D-TGCT is a locally aggressive process with a high recurrence rate of around 40–60% [12, 33]. MRI is fundamental to assess D-TGCT intra- and extra-articular extension and can help avoid incomplete resections.

The knee joint intracapsular space is composed of multiple and interconnected synovial recesses, some of which are rarely apparent on MRI of a healthy, non-affected knee [34]. D-TGCT is characteristically found in certain areas showing a reproducible distribution pattern similar to loose bodies [35]. Therefore, D-TGCT lesions can be identified on MRI due to reactive joint effusion making synovial recesses apparent and due to signal intensity contrast between tumour and fluid.

The same MRI protocol should be used for pre-and post-treatment assessment (as described in Additional file 1: Table S1)*.* Assessment of joint recesses may be done first in the sagittal plane (comparing T1-WI with T1-WI fat-suppressed (FS) gadolinium (Gd) side-to-side for anatomy and enhancement of lesions). Secondly, side-to-side comparison of axial fluid sensitive sequences (we use T2 DIXON) and T1-WI FS Gd images helps distinguish tumour from (rim-)enhancing synovial fluid or cyst-like components. Coronal images add value for assessment of erosions and femorotibial chondropathy.

The following areas should be carefully scrutinised and reported (Fig. 1):

The *anterior compartment:*

1. The suprapatellar recess is localised between the prefemoral and suprapatellar fat pads. It is the most distensible synovial recess, often containing hemosiderin deposits along its posterior synovial surface and nodular proliferations surrounded by joint fluid. Simultaneous assessment of T1-weighted images and fluid-sensitive sequences in axial and sagittal planes is crucial. The prefemoral fat has well-defined rounded margins, but not infrequently; it acquires dendritic borders protruding into the suprapatellar pouch resulting in TGCT overestimation due to taking fat-suppressed adipose tissue for TGCT.

The medial and lateral parapatellar gutters are key areas to assess on axial images because D-TGCT is frequently trapped here.

2. The infrapatellar synovial recess is divided into the superior and inferior recesses, orientated vertically and horizontally, respectively, including the localisation of tumour underneath the anterior horns of the menisci and the intermeniscal ligament.
3. The involvement of pre-patellar and infra-patellar bursae in D-TGCT is rare and should urge the investigation of other entities such as gout.

The *posterior compartment* has a limited distensible capacity defined by the posterior femoral capsule:

1. The subgastrocnemius synovial recesses, also referred to as retrocondylar or posterior femoral recesses, are localised underneath the gastrocnemius head insertions [35]. These areas present as fat signal intensity triangles with concave margins on sagittal T1-weighted images. Subgastrocnemius collapsed reactive synovitis without intervening effusion results in D-TGCT overestimation. Replacement of fat by low signal intensity tissue is in keeping with hemosiderin. Ganglion cysts at the medial or lateral gastrocnemius origins may be indistinguishable from tumour, because D-TGCT tends to show multiloculated thick-walled cystic masses [36]. The cleft-like morphology and presence of hemosiderin point towards D-TGCT, where it is prone to develop extra-articular extension encasing the gastrocnemious tendons.
2. The PCL recess is located between the PCL and the posterior joint capsule. It usually harbours fibrofatty tissue with thin enhancing septa, which can be misdiagnosed as TGCT on fat-suppressed sequences [23]. The inspection of T1-weighted images and the absence of mass effect (posterior femoral capsule bulging) confirms its nature. This location is typical for extra-capsular D-TGCT popliteal extension due to perforating vessels on the posterior femoral capsule [37].
3. The subpopliteus synovial recess encases the popliteus tendon. It is connected to the popliteal hiatus between the posterosuperior and anteroinferior popliteomeniscal fascicles. It extends behind the posterior horn of the lateral meniscus to sit underneath the popliteus muscle belly in contact with the posterior aspect of the tibia. The subpopliteal recess represents a continuum with the proximal tibiofibular joint in approximately 10% of patients [38].
4. The Baker’s or popliteal cyst is also known as the medial gastrocnemius—semimembranosus bursa. Baker’s cyst D-TGCT involvement is considered intra-articular extension because of the communication with the knee joint synovial lining. Due to its dependent location and ball-valve communication mechanism, a Baker’s cyst can harbor multi-cystic proliferations with or without involvement of another compartment. The medial gastrocnemius-semimembranosus tendon cross-over may mimic haemorrhage deposition, but its anti-gravitational site and axial tracking differentiate it from hemosiderin deposition.
5. Some bursae, such as the semimembranosus-tibial collateral ligament bursa (semimembranosus bursa) and pes anserinus are not connected to the knee joint and are atypical locations for D-TGCT.

The *middle compartment* is a tight area where distension is limited.

1. The central synovial recess is situated in front of the ACL.
2. The intercruciate space is challenging to assess on MRI, as D-TGCT is generally underestimated in this area due to the similar low signal intensity compared to the cruciate ligaments. Intra-articular pericruciate ganglion cysts should be differentiated from tumour. Bone erosions caused by the tumour in the intercondylar notch can be undercalled as cystic change at the insertion of the cruciate ligaments, which is a common finding on normal knee MRIs [39]. Bone erosions in D-TGCT typically contain enhancing tumour tissue (Fig. 2C, E).
3. The femorotibial synovial recesses, both medial and lateral, are occupied in severe D-TGCT cases when tumour volume generally exceeds 80–100 cc. These areas are prone to develop cortical erosions as they are virtually not distensible.

MRI is the modality of choice to assess residual D-TGCT after synovectomy and for postoperative follow-up. Common postoperative changes include skin thickening, fat stranding or inflammation in Hoffa (in the area of arthroscopy portal entrances), subcutaneous and intramuscular oedema with susceptibility artefacts secondary to surgical clips (Fig. 2B). Joint effusion may be reduced due to drainage. Diffuse synovial thickening is equivocal for D-TGCT residual disease within the first six months because of associated reactive synovitis [40]. Growing enhancing solid and nodular synovial thickening should raise the suspicion of disease recurrence (Fig. 2B, E).

### Systemic therapy response evaluation

With the arrival of systemic therapies targeting CSF1/CSF1R in D-TGCT patients not regarded amenable to surgery, MRI is required to objectively assess treatment response [16, 17, 41]. Quantification of change in tumour volume is the main feature to evaluate the response of these agents. To date, Response Evaluation Criteria in Solid Tumours (RECIST) 1.1 is the most frequently used tool to detect a change in tumour size by calculating the sum of the longest diameters for all target lesions. In addition, a modification of RECIST (m-RECIST) can be applied, adding a short axis measurement for target lesions, offering higher accuracy [17]. However, the irregular tumour shape, absence of nodular lesions, asymmetrical growth, variable enhancement after contrast, and lack of clear tumour margins make it challenging to apply linear measurements [42].

Peterfy et al. developed a semiquantitative, joint-specific, visual tumour volume score (TVS) for D-TGCT. This score was developed analogous to and based on arthritis visual scores used in clinical trials. TVS expresses tumour volume as a percentage of the estimated volume of the maximally distended normal synovial cavity of the involved joint. TVS can incorporate all tumour regions and defines tumour size relative to the joint size [42]. However, since TVS is a semiquantitative tool, clinicians have to estimate the percentage of tumour volume, limiting its reproducibility. In addition, TVS has not been validated as a method for response assessment yet. Therefore, there is an urgent need for an automated tool measuring D-TGCT tumour volume on MRI.

**Fig. 7:**
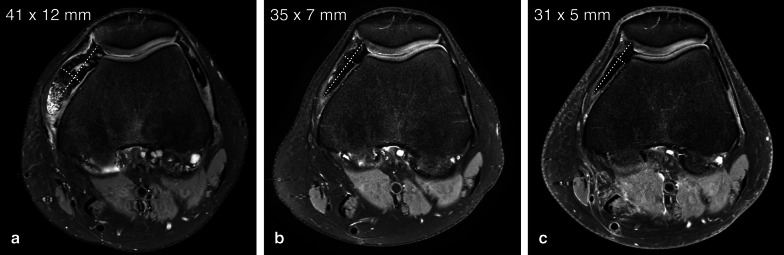
MRI findings after treatment of D-TGCT with a CSF1R inhibitor. **a** Axial PD SPAIR images in a patient with intra-articular D-TGCT. Baseline image showing a target lesion (two axial diameters measured according to modified RECIST, dotted lines) in the medial suprapatellar recess. Note a smaller similar lesion in the lateral suprapatellar recess. **b** After 8 weeks on CSF1R-inhibitor therapy, the tumour showed a significant decrease in size and signal intensity. Joint effusion was resolved (not shown). The patient experienced improvement in symptoms of pain and swelling. **c** After 36 weeks, residual low signal intensity hemosiderin “scars” remained both in the medial and lateral suprapatellar recesses

Aside from changes in tumour size, other specific MRI findings following CSF1R inhibitors have been described in pilot studies. These findings include a decrease in signal intensity on fluid-sensitive sequences with a reduction of capsular distension and joint effusion and an increase in hemosiderin deposition [43]. Decreased enhancement seems to be an equivocal parameter. These imaging features appear to correlate well with clinical improvements, such as pain reduction (Fig. 7) [44].

Of note, patients may experience complete symptomatic relief even in the setting of what appears to be an incomplete response based on imaging [43]. So-called hemosiderin scars remain as a low signal intensity rim lining the synovium after therapy, without corresponding clinical complaints. It has been suggested to use “complete response” in case of residual hemosiderin scars with a short axis < 5 mm, however this approach needs further study [42].

### Future directions: 3D segmentation

Following the limitations of the current response criteria, volumetric quantification techniques integrated with Artificial Intelligence (especially deep learning) methods may help measure tumour change. However, developing such an automated quantification method is challenging due to irregular growth and heterogeneous signal intensity of TGCT [42].

We performed a first step towards objective volumetric quantification of D-TGCT by segmenting 3D tumour volume in 40 treatment-naïve patients with D-TGCT. All imaging data were anonymised, and informed consent was waived by the institutional review board (G19.127). Measuring tumour volume was done in Brainlab (Elements), an application that is commonly used for preoperative planning of bone and soft-tissue tumours.

**Fig. 8:**
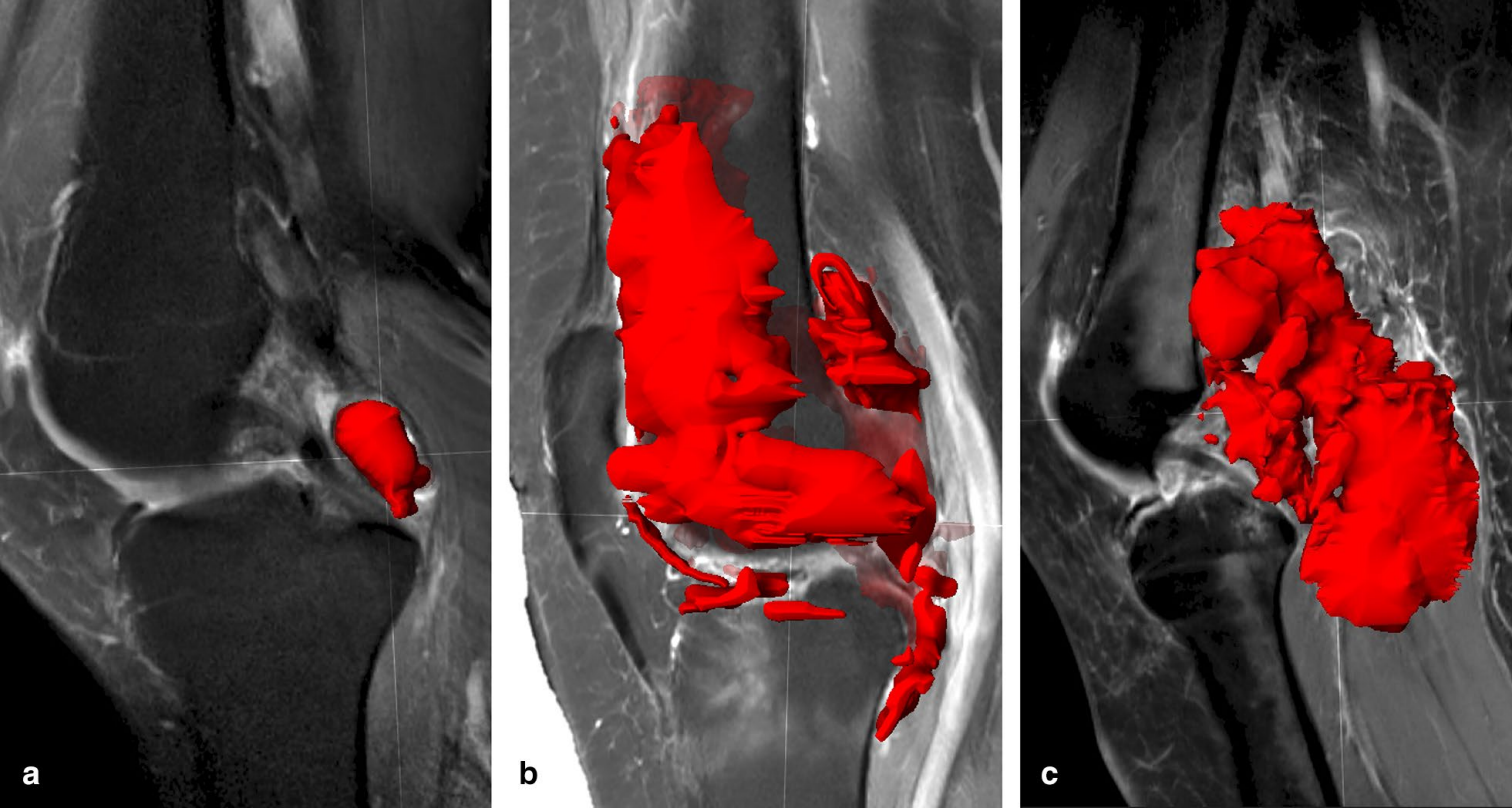
Volumetric segmentations of D-TGCT performed with Brainlab software on sagittal T1 SPIR-weighted sequences post gadolinium. **a** Tumour is segmented in the PCL recess (volume shown in red). Volume: 3.8 cm^3^. **b** Tumour segmentation in anterior, middle and posterior compartments. Volume: 86.2 cm^3^. **c** Tumour segmentation of a case with marked posterior disease, present in the PCL recess, subgastrocnemius synovial recesses, Baker’s cyst, and extending extra-articular in the popliteal fossa. Volume: 91.9 cm^3^

For 3D segmentation, a sagittal T1 spectral inversion recovery (SPIR) Gd and an axial T2 DIXON (water only) scan were utilised. In addition to the total volume, the volumes in the anterior and posterior compartments of the knee were calculated separately. The mean total volume was 44 cm^3^ (range 3.2–208.8 cm^3^). The mean volume located in the anterior compartment was 21 cm^3^ (range 0.0–205.8 cm^3^) and in the posterior compartment 23 cm^3^ (range 0.0–172.9 cm^3^). Examples of volumetric segmentation in three different patients are shown in Fig. 8.

We showed that 3D MR segmentation allows to objectively quantify the tumour burden of TGCT and provides a quick visual assessment of TGCT lesion distribution throughout the knee. However, since 3D segmentation of TGCT is time-consuming and operator-dependent, future steps will focus on automating this process.

## Conclusions

MRI is the modality of choice in diagnosing D-TGCT, providing preoperative mapping and assessment of response to systemic therapies. However, due to its irregular shape, extensive growth and low signal intensity, D-TGCT disease extent can be challenging for the radiologist. We highlighted imaging characteristics of D-TGCT affecting the knee and provided a structured report template (Table 1). In addition, pitfalls such as mimickers of D-TGCT were addressed, and evaluation of tumour response following new systemic therapies. Finally, we demonstrated a first step towards objective 3D volume quantification of D-TGCT. Automated quantification of tumour load to assess treatment response will become more important as systemic medical therapies evolve quickly.

**Table 1:** D-TGCT Knee MRI structured report template

Items	Findings
1. Shape	Well-circumscribed nodules *and /or* diffuse villous synovial thickening
2. Site	
2a. Intra-articular	Anterior compartment (suprapatellar, superior and inferior infrapatellar recesses) Middle compartment (central, femorotibial, intercruciate recesses) Posterior compartment (retrocondylar, PCL, subpopliteus, Baker’s cyst) Cartilage and bone invasion by the tumour (tumour extension in pressure erosions)
2b. Extra-articular	Posterior transcapsular extension to popliteal fossa Muscular-tendinous involvement (invasion or encasement > 180°) Ligament involvement (invasion or encasement > 180°) Neurovascular bundle involvement (> 180° encasement of the tibial nerve and/or popliteal artery/veins)
3. Signal intensity	T1-WI: (hypo-/isointense) and (homogeneous/heterogeneous) T2-WI FS: (hypo-/iso-/hyperintense) and (homogeneous/heterogeneous) T1-WI FS Gd: enhancement (absent/present) and (homogeneous or heterogeneous) T2*-WI or T1 GRE: blooming (absent/present)
4. Size	Bidimensional measurements [RECIST 1.1: long axis of target lesions, modified RECIST: long and short axis of target lesions] Volumetric tumour burden (Tumour Volume Score)
5. Secondary findings (complications)	Joint Effusion (> 10 mm anteroposterior in the suprapatellar pouch) Reactive synovitis (sometimes fatty metaplasia, hyperintense on T1-WI) Secondary osteoarthritis/Chondromalacia
Conclusion	
Subtype (growth pattern)	Diffuse-type TGCT (≥ 2 synovial recesses)
Extension	Intra-articular and/or extra-articular
Complications	Secondary osteoarthritis (mild, moderate, severe)

PCL, posterior cruciate ligament; WI, weighted imaging; FS, fat-suppressed; Gd, gadolinium; GRE, gradient echo; RECIST, response evaluation criteria in solid tumours

## Supplementary Information

**Additional file 1: Table S1.** MRI protocol for D-TGCT of the knee.

## Data Availability

Not applicable.

## Abbreviations

ACL: Anterior cruciate ligament
ADC: Apparent diffusion coefficient
Ax: Axial
Cor: Coronal
CSF1: Colony-stimulating factor 1
CSF1R: Colony-stimulating factor 1-receptor
CT: Computed tomography
D-TGCT: Diffuse-type tenosynovial giant cell tumour
DWI: Diffusion-weighted images
FFE: Fast field echo
FOV: Field-of-view
FS: Fat-suppressed
Gd: Gadolinium
GRE: Gradient echo
IR: Inversion recovery
L-TGCT: Localised-type tenosynovial giant cell tumour
m-RECIST: Modification of RECIST
MRI: Magnetic Resonance Imaging
MST: Multi-stack
PCL: Posterior cruciate ligament
PD: Proton density
RECIST: Response evaluation criteria in solid tumours
Sag: Sagittal
SE: Spin-echo
SP(A)IR: Spectral (adiabatic) recovery
TFE: Turbo field echo
TGCT: Tenosynovial giant cell tumour
TSE: Turbo spin echo
TVS: Tumour volume score
WI: Weighted imaging
