# Supplementary material for: MRI of diffuse-type tenosynovial giant cell tumour in the knee: a guide for diagnosis and treatment response assessment
Source: Insights Imaging. 2023 Feb 1;14:22. doi: 10.1186/s13244-023-01367-z (PMC9892412; doi:10.1186/s13244-023-01367-z)
Supplement: Supplementary file 1 — Additional file 1: Table S1. MRI protocol for D-TGCT of the knee. [file 13244_2023_1367_MOESM1_ESM.pdf]

<u>ELECTRONIC SUPPLEMENTARY MATERIAL</u>

MRI of diffuse-type Tenosynovial Giant Cell Tumour in the knee: a guide for diagnosis and treatment response assessment

Insights Imaging (2023) Spierenburg G, Suevos Ballesteros C, Stoel BC et al.

**Scan**	**Name**	**Technique**	**FOV**	**Thickness (mm)**	**Slices**	**Scan time**
**1**	Survey Ax	T1FFE				0:10
**2**	Survey MST	T1FFE				0:50
**3**	T1 Sag	TSE	220x180	3,0	50	3:30
**4**	PD SPAIR Sag	TSE	220x180	3,0	50	4:13
**5**	PD Cor	TSE	220x180	3,0	50	2:45
**6**	T2 DIXON Ax	TSE	180x180	4,0	40	2:51
**7**	T1W FFE Sag #	FFE	220x180	3,00	50	4:30
**Administer contrast agent during dynamic scan: 0,2 ml Clariscan per kg body weight**						
**8**	Dynamic + T1 map *	3D T1 TFE	250	5,0 – 10,0	9	5:09
**9**	T1 SPIR Gd Sag	TSE	220x220	3,0	50	3:54
**10**	T1 SPIR Gd Ax	TSE	180x180	4,0	40	1:55

Supplementary table 1. MRI protocol for D-TGCT of the knee.

Parameters shown are for a 3 Tesla Ingenia MRI scanner, Philips, Eindhoven, The Netherlands.

FOV Field-of-view; *Ax* Axial; *FFE* Fast Field Echo; *MST* multi-stack; *Sag* Sagittal; *TSE* Turbo Spin Echo; *SP(A)IR* Spectral (Adiabatic) Inversion Recovery; *PD* Proton Density; *Cor* Coronal; *Gd* Gadolinium; *TFE* Turbo Field Echo

* Addition of a dynamic sequence post-contrast is optional.

# Gradient echo sequence for assessment of blooming artifact. This may be replaced by a T2* weighted sequence.
